# TRIM32 biallelic defects cause limb-girdle muscular dystrophy R8: identification of two novel mutations and investigation of genotype–phenotype correlation

**DOI:** 10.1186/s13395-023-00319-x

**Published:** 2023-05-22

**Authors:** Yuqing Guan, Xiongda Liang, Wei Li, Wanying Lin, Guanxia Liang, Hongting Xie, Yu Hou, Yafang Hu, Xuan Shang

**Affiliations:** 1 Department of Neurology, Nanfang Hospital, Southern Medical University, Guangzhou, China; 2 Department of Medical Genetics, School of Basic Medical Science, Southern Medical University, Guangzhou, China; 3 Department of Laboratory Medicine, Nanfang Hospital, Southern Medical University, Guangzhou, China; 4 Department of Hematology, Southwest Hospital, Third Military Medical University, Chongqing, China; 5 Innovation Center for Diagnostics and Treatment of Thalassemia, Nanfang Hospital, Southern Medical University, Guangzhou, China; 6 Guangdong Provincial Key Laboratory of Single Cell Technology and Application, Guangzhou, China

**Keywords:** TRIM32, LGMD R8, Limb-girdle muscular dystrophy

## Abstract

**Background:**

Limb-girdle muscular dystrophy R8 (LGMD R8) is a rare autosomal recessive muscle disease caused by TRIM32 gene biallelic defects. The genotype–phenotype correlation of this disease has been reported poorly. Here, we report a Chinese family with two female LGMD R8 patients.

**Methods:**

We performed whole-genome sequencing (WGS) and Sanger sequencing on the proband. Meanwhile, the function of mutant TRIM32 protein was analyzed by bioinformatics and experimental analysis. In addition, a summary of the reported TRIM32 deletions and point mutations and an investigation of genotype–phenotype correlation were performed through a combined analysis of the two patients and other cases reported in previous literature.

**Results:**

The two patients displayed typical symptoms of LGMD R8, which worsened during pregnancy. Genetic analysis by whole-genome sequencing (WGS) and Sanger sequencing showed that the patients were compound heterozygotes of a novel deletion (chr9.hg19:g.119431290_119474250del) and a novel missense mutation (TRIM32:c.1700A > G, p.H567R). The deletion encompassed 43 kb and resulted in the removal of the entire TRIM32 gene. The missense mutation altered the structure and further affected function by interfering with the self-association of the TRIM32 protein. Females with LGMD R8 showed less severe symptoms than males, and patients carrying two mutations in NHL repeats of the TRIM32 protein had earlier disease onset and more severe symptoms than other patients.

**Conclusions:**

This research extended the spectrum of TRIM32 mutations and firstly provided useful data on the genotype–phenotype correlation, which is valuable for the accurate diagnosis and genetic counseling of LGMD R8.

**Supplementary Information:**

The online version contains supplementary material available at 10.1186/s13395-023-00319-x.

## Background

Neuromuscular diseases (NMDs) are a broad and heterogeneous collection of disorders involving peripheral nerve and muscle dysfunction. Limb-girdle muscular dystrophy (LGMD) is the fourth most common genetic NMD that primarily affects skeletal muscles, resulting in progressive proximal muscle weakness caused by the loss of muscle fibers [1]. According to the current classification, there are 31 types of LGMD: 5 dominant subtypes (LGMD D1-D5) and 26 recessive subtypes (LGMD R1-R25 and R, pending). Each subtype is associated with unique gene mutations, and there is significant heterogeneity in disease expression, progression and prognosis in the different subtypes [2]. It is estimated that the prevalence of LGMD is between 1 in 14,500 and 1 in 123,000 for all subtypes, with a carrier frequency of 1:211, but the prevalence of LGMD varies by region of the world [3].

LGMD R8 (previously named LGMD 2H) is caused by mutations in the TRIM32 gene encoding the TRIM32 protein, an E3 ubiquitin ligase [4, 5]. It is a slowly progressive disease characterized by proximal muscle weakness, atrophic wasting of the lower extremities and mild to moderate elevation of creatine kinase (CK) levels. Facial weakness, pterygoid scapulae, hypertrophic calves, and contracted Achilles tendons are not uncommon [6]. The clinical manifestations widely range from nearly asymptomatic to a more severe wheelchair-bound phenotype [7]. The age of onset is defined as the age at which the first muscle weakness was noticed by the patients or parents, which is an important risk factor associated with disease progression [8].

The TRIM32 gene encodes a protein of 653 amino acids [9]. The N-terminal conserved motif of the TRIM32 protein consists of a RING structural domain, a B-box and a coiled-coil structural domain, while its C-terminal part consists of six NHL repeats. The RING domain is mainly associated with TRIM32 E3 ubiquitin ligase activity, the B-box and coiled-coil domains are involved in proper protein folding, and the C-terminal domain mainly mediates TRIM32 self-association and interactions with its substrates [10]. Most mutations causing LGMD R8 are located in the domain of the NHL repeat sequence, while mutations in the domain of the B-box cause another disease called Bardet-Biedl syndrome (BBS). This disease is characterized by obesity, retinitis pigmentosa, polydactyly, renal abnormalities, learning disabilities, and hypogonadism, without the symptoms of myotonic dystrophy, which may imply different pathogenic mechanisms from LGMDR8 [11].

The first mutation (TRIM32:c.1459G > A, p.D487N) causing LGMD R8 was identified in the Hutterite population of North America [12]. Homozygotes of this missense mutation showed slowly progressive symptoms of proximal muscle weakness and wasting [13]. To date, fewer than 100 patients with this disease have been reported worldwide, most of which are distributed in the Hutterite population, and the prevalence is extremely low. Only one case of homozygous deletion of 2 kb was reported in China [14].

Here, we identified two patients in a Chinese family with clinical symptoms of LGMD R8 who were genetically confirmed to be compound heterozygotes for a novel missense mutation (TRIM32:c.1700A > G, p.H567R) and a novel TRIM32 deletion (chr9.hg19:g.119431290_119474250del). We also present a summary of relevant literature on the mutation spectrum of the TRIM32 gene. Moreover, to help clinicians had better recognize this disease, we performed the first investigation of the genotype–phenotype correlation of known LGMD R8 patients.

## Patient and method

### Patients

**Fig. 1:**
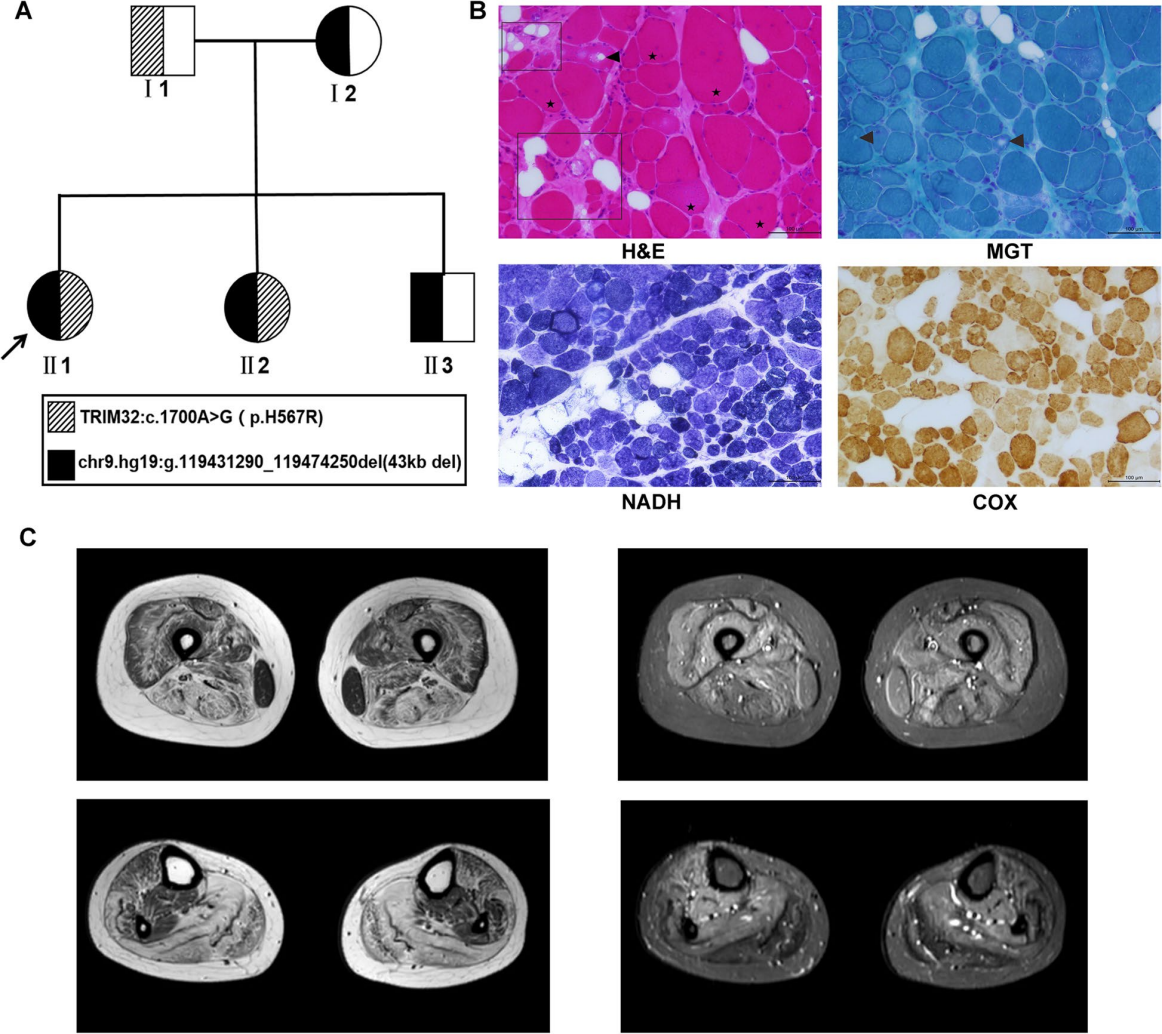
Pedigree data of the family and clinical phenotype. **A** Pedigree data of the family; the proband is marked with an arrow. **B** Representative images of patient muscle biopsy are shown. H&E staining showed vacuoles inside muscle fibers (arrow), interstitial fat infiltration and connective tissue hyperplasia (inside rectangular box), and multiple internalized nuclei in muscle fibers of various size (star). MGT staining showed vacuoles inside muscle fibers (arrow). NADH and COX staining showing irregular internal architecture with patchy light-stained areas. **C** Magnetic resonance imaging (MRI). Axial T1-weighted and T2-weighted MRI scans of the thigh and calf

The proband (II1, Fig. 1A) was a 30-year-old woman from Henan Province in southern China. She felt generally fatigued and was experiencing weakness of all limbs during pregnancy and came to the Department of Neurology, Nanfang Hospital (Southern Medical University), for consultation. The neurologist took a detailed medical history and performed neurologic examinations and routine laboratory examinations. After these clinical examinations, she was clinically diagnosed as limb-girdle muscular dystrophy. Neuromuscular disease V3-Panel next-generation sequencing (NGS, MyGenostics, Beijing, China) was performed for identification of the disease-causing gene, and the family members were referred to our laboratory for further research. Blood samples were collected from the patient and her family members after obtaining informed consent.

### Whole-genome sequencing (WGS)

Whole-genome sequencing (WGS, Novogene, Beijing, China) analysis was used to predict the range of the novel deletion in the proband. First, the genomic DNA sample was fragmented by sonication to a size of 350 bp. Then, DNA fragments were end-polished, A-tailed, and ligated with the full-length adapter for Illumina sequencing, followed by further PCR amplification. After the PCR products were purified by an AMPure XP system (Beckman Coulter, Beverly, USA), and libraries were analyzed for size distribution by NGS3K/Caliper and quantified by real-time PCR (3 nM). After cluster generation, the DNA libraries were sequenced on an Illumina platform, and 150 bp paired-end reads were generated. Validated sequencing data were aligned to the reference genome (human_37) by BWA [15] and Samblaster (Faust, G.G. and Hall, I.M.) to obtain the initial alignment results in BAM format. Then, the results of the comparison were sorted using SAMtools [16], and the duplicate reads were marked using Samblaster. Finally, control-FREEC [17] software was used to detect CNVs by examining the depth distribution of reads of the samples on a reference genome.

### Sanger sequencing

The TRIM32 gene was amplified by PCR, and the PCR products were subjected to full-length Sanger sequencing to verify the variants identified by NGS (F1: 5′-GGGCATGAATACTGTGCTGT-3′; R1: 5′-GCTGGAATCAGGACATAGGCT-3′).

One pair of primers was designed upstream and downstream of the deletion site for Gap-PCR, and the PCR products were subjected to Sanger sequencing to verify the large fragment deletion identified by WGS and to clarify the break site of the deletion.

(F2: 5′-GAATCCAAGTCTGGAACAAGTATG-3′; R2: 5′-GTGGTGGTAAGATAAACCTAGAAA-3′; F3: 5′-AGCCAACATCTTCAACAAATAACA-3′; R3: 5′-GGAGGTATGCTGGGCATTTATTAT-3′).

### Plasmid construction and cell transfection

The extensive coding sequence of TRIM32 was acquired from healthy individual complementary DNA (cDNA) and cloned into the MSC site (ECOR I and Xma I) of the green fluorescent pEGFP-N1 vector and PHAGE vector with flag tag to construct the wild-type expression vector pEGFP-N1-TRIM32-WT and PHAGE-TRIM32-WT-Flag. Next, two pairs of complementary primers were used to construct four mutant expression vectors pEGFP-N1-TRIM32-D487N, pEGFP-N1-TRIM32-H567R, PHAGE-TRIM32-D487N-Flag, and PHAGE-TRIM32-H567R-Flag with p.D487N and p.H567R mutations by site-directed mutagenesis. The primer sequences used to introduce the mutations were 5′-AGTTTGTAGTAACCAATGTGGAAGGTGGAA-3′ for p.D487N and 5′-TCGCCAGATTAGCCGCTTCTTCTCGGAGAA-3′ for p.H567R. Finally, the four resulting mutant expression vectors were sequenced to verify the success of the site-directed mutagenesis.

HEK-293 T cells were cultured in 10% fetal bovine serum (FBS) and 90% DMEM (Gibco) at 37 °C and 5% CO2. Then, 3 μg of wild-type pEGFP-N1-TRIM32-WT and PHAGE-TRIM32-WT-Flag plasmid and mutant pEGFP-N1-TRIM32-D487N, pEGFP-N1-TRIM32-H567R, PHAGE-TRIM32-D487N-Flag and PHAGE-TRIM32-H567R-Flag plasmids were transiently transfected into HEK-293 T cells by using Lipofectamine 2000 (Thermo Fisher Scientific). The pEGFP-N1 plasmids expression of the plasmids was analyzed using confocal microscopy 48 h after transfection.

### Protein purification and western blot

Flag-tagged TRIM32 and mutants were transfected into HEK-293 T cells as described above. After 48 h, the cells were lysed in NP-40 Lysis Buffer (Beyotime, Shanghai, China) and TRIM32 was purified by immunoaffinity chromatography using Anti-FLAG (DYKDDDDK) -Sc Immunomagnetic Beads (Daian Biotech, Wuhan, China). Purified proteins were separated by SDS–PAGE and electro blotted onto nitrocellulose membranes. The membranes were incubated overnight at 4 °C with rabbit anti-TRIM32 antibody (Abcam, UK). Next, the membranes were incubated for 2 h with goat anti-rabbit IgG (Abcam, UK). Protein expression was detected by exposure to an automatic chemiluminescence image analysis system (Tanon, Shanghai, China).

### RNA isolation and quantitative analysis

Total RNA was extracted from the peripheral blood using TRIzol reagent (Accurate Biotechnology, Hunan, China). Then, RNA was reverse-transcribed to cDNA using the Evo M-MLV RT Kit with gDNA Clean for qPCR II (Accurate Biotechnology, Hunan, China). To assay the expression level of TRIM32 mRNA in peripheral blood, q-PCR was performed by LightCycler®96 (Roche, USA) using SYBR Green as the reporter molecule and GAPDH as the housekeeping gene. Relative quantitative comparison of ΔΔCT was used to calculate the difference in gene expression (F4: 5′-AGGGCATGAATACTGTGCTGT-3′; R4: 5-AGATGGTATGGCCACAGTGC-3′).

### Bioinformatics analysis

To further understand the effect of mutant TRIM32 protein on function or structure, we studied the mutated amino acid sequence and analyzed its physicochemical properties, sequence conservation, 3D structure prediction. Species conservation analysis of mutant site bases and amino acids in several similar species was performed using Clustal X. The 3D models of wild-type and mutant TRIM32 proteins were constructed by I-TASSER [18]. The generated PDB file was imported into PyMol software to compare and analyze the 3D structure of the protein. The damaging effect of amino acid sequence variants on protein function was predicted by PolyPhen-2 software [19].

### Literature review

We conducted an electronic literature search in the PubMed database using “TRIM32”, “LGMD”, “LGMD 2H” or “LGMD R8”, “neuromuscular disease” and “mutation” as keywords and identified all potentially relevant articles, limited to those in English. All articles were reviewed, and TRIM32 mutations and cases with detailed clinical and genotype information were collected and analyzed.

### Statistical analysis

Statistical differences between groups were determined by an unpaired *T* test. Multiple comparisons of the six groups on region class were also carried out through multiple independent unpaired *T* tests between the two groups. The quantified results are shown as the mean ± SD. Statistical significance was determined by a *P* value of less than 0.05. All statistical analyses were performed using the statistical software GraphPad Prism 7.

## Results

### Two female patients presented typical LGMD symptoms

The proband (II1) was born from healthy Chinese parents. She had normal motor developmental milestones, but her sports performance was worse than that of her peers. Physical examination showed normal intelligence and cranial nerve signs. Neurological examination revealed muscle atrophy of the bilateral proximal and distal lower extremities. Muscle strength (Medical Research Council grade scale) is shown in Table 1. She had a positive Gowers’ sign and a mixture of stoppage and waddling gait. She could not perform toe walking or heel walking. The knee reflex and Achilles tendon reflex were absent on both sides. The serum CK level was mildly elevated to 610 IU/L (normal range 2–178 IU/L). Nerve conduction studies (NCSs) showed normal results. Electromyography (EMG) revealed myopathic changes with the duration of motor unit action potentials (MUAPs) of the biceps brachialis, vastus medialis, and tibialis anterior decreased by 21%, 30%, and 27% respectively, and mean amplitude of MUAPs of the above muscles being 305 μV, 363 μV, and 332 μV respectively (reference value 308 μV, 350 μV, and 381 μV). Abnormal spontaneous activities were not inspected. Muscle biopsy showed myopathic features, including muscle fiber size variation, atrophic and hypertrophic fibers, increased interstitial fat and connective tissue, increased internalized nuclei and scattered vacuolar changes inside some muscle fibers (Fig. 1B). Thigh and lower leg magnetic resonance imaging (MRI) revealed symmetric changes in muscle atrophy and fatty infiltration, which were more remarkable in the biceps femoris, semitendinosus, semimembranous, and gastrocnemius, with the gracilis, tibialis posterior, and flexor digitorum longus markedly spared (Fig. 1C).

**Table 1:** Clinical and genetic information of family members

Patient ID		I1	I2	II1	II2	II3
Age of onset (years)		n.r	n.r	24	27	n.r
Sex		M	F	F	F	M
Height (cm)		162	150	153.5	149.5	177
Weight (kg)		52	52.5	38.2	39.7	80
Muscle strength (MRCs)	Neck flexion	n.d	n.d	5/5	5/5	n.d
	Neck extension			5/5	5/5	
	Shoulder abduction			4/5	4/5	
	Elbow extension			3/5	3/5	
	Elbow flexion			4/5	4/5	
	Grasp			4/5	4/5	
	Hip flexion			3/5	4/5	
	Knee extension			4/5	5/5	
	Knee flexion			4/5	5/5	
	Foot dorsal extension			3/5	4/5	
	Foot plantar flexion			3/5	4/5	
CK(U/L) (reference range 2–178 U/L)		normal	normal	610↑	427↑	Normal
Genotype(s)		p.H567R/N	43 kb deletion/N	p.H567R/43 kb deletion	p.H567R/43 kb deletion	43 kb deletion/N

*F* female, *M* male, *MRC* Medical Research Council grade scale (scaled from 0 to 5 by severity), *CK* creatine kinase, ↑ increased, *n.d.* not done, *n.r.* not reported

Patient 2 (II2) is the younger sister of patient 1. She has similar poor performance in sports as her sister. Both sisters have a short and thin habitus. Muscle strength examination revealed similar results to her sister, with the proximal and distal muscles of the upper and lower limbs affected (Table 1), and she had a positive Gowers’ sign and identical stoppage and waddling gait. Serum CK was mildly elevated to 427 IU/L. EMG revealed myopathic MUAPs in the biceps brachialis, vastus medialis, and tibialis anterior. The other family members (I1, I2, and II3) all showed a normal phenotype (Table 1).

Two patients (II1 and II2) both experienced progressive weakness and fatigue during pregnancy. The proband was pregnant for the first time at the age of 18. During this pregnancy, she felt generally fatigued, and she unusually lost 4 kg during the pregnancy without any known reason, but she did not showed typical LGMD symptoms at that time. Then, months after the delivery, she returned to her pre-pregnancy status. She was pregnant for the second time at the age of 23, and the fetus died at the seventh month of pregnancy without an apparent cause. During her third pregnancy at age 24, she felt weakness of all limbs, which was more severe in the lower limbs and she had to push her knees with hands when standing up from a squatting position. Her weakness slowly worsened during the latter part of the pregnancy until it was terminated by cesarean section, and she gave birth to a healthy boy. The weakness did not improve after delivery, nor did it get worse until she was pregnant for the fourth time at age 29. Then, she felt the deterioration and weakness along with chest tightness and palpitations. The pregnancy was terminated by induced abortion. Her sister had an uneventful first pregnancy at age 23 and experienced progressive weakness and fatigue during her second pregnancy at age 27. After delivery at age 28, her symptoms reached a plateau. The two patients have been followed up for 5 years up to present. Neither of them got pregnant again during this period, and their motor function remains stable.

### A novel missense mutation and a novel deletion of the TRIM32 gene were identified

Analysis of neuromuscular disease V3-panel NGS showed that the proband was a homozygote of a novel missense mutation (TRIM32:c.1700A > G, p.H567R), and copy number analysis of the TRIM32 gene by RT-PCR revealed that one allele was deleted. Therefore, the proband (II1) should be a compound heterozygote of a missense mutation and a deletion. Genetic analysis showed that the missense mutation was inherited from her father (I1), and the deletion was inherited from her mother (I2). Another female patient (II2) had the same genotype as II1, and her brother (II3) only inherited the deletion from the mother (Fig. 1A).

**Fig. 2:**
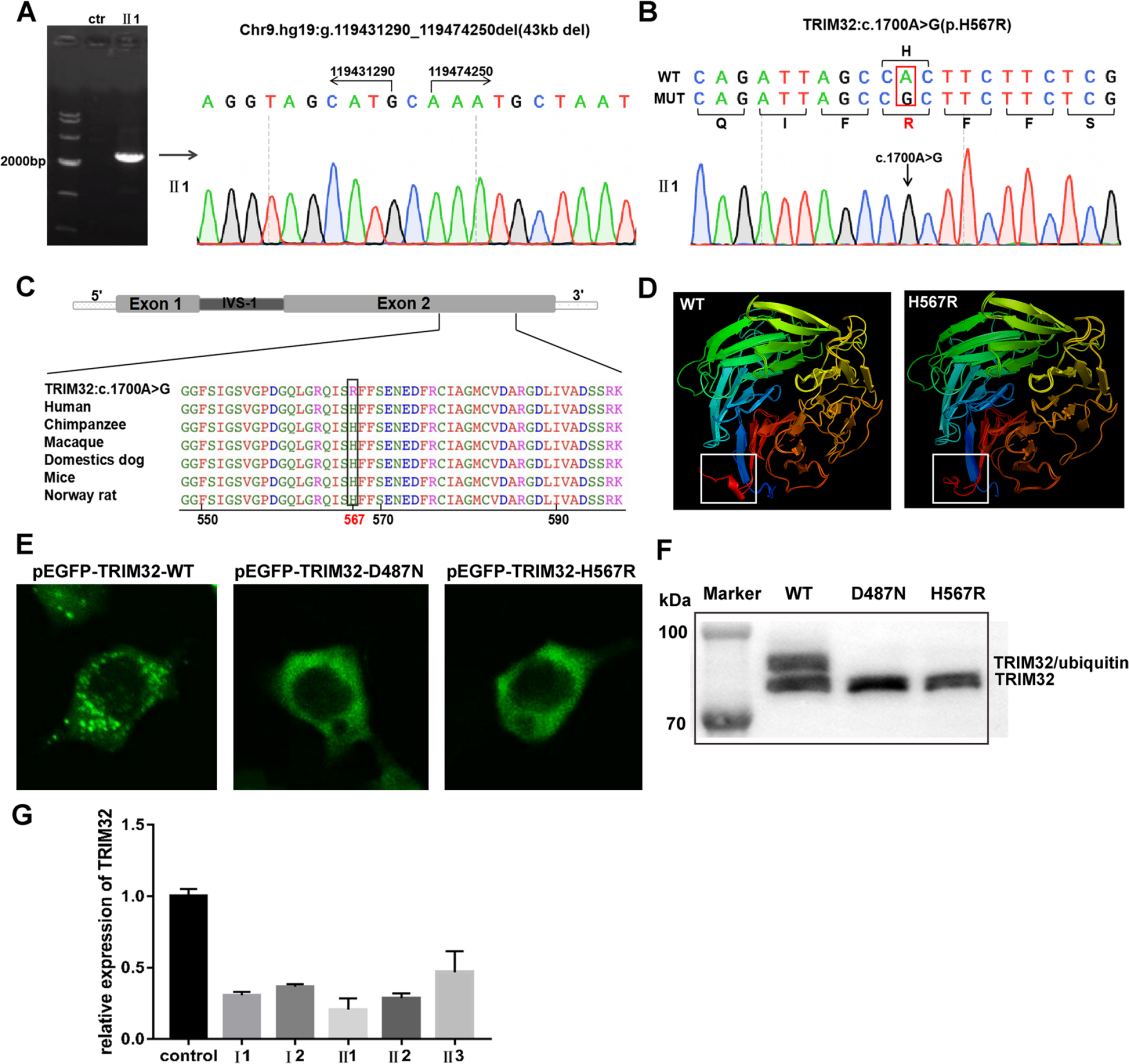
Molecular genotyping and expression analysis of mutated TRIM32 at the RNA and protein levels in vivo and in vitro. **A** Detection of the deletion in TRIM32 by multiplex gap-PCR and the exact deletion breakpoint of TRIM32 in the proband (chr9.hg19:g.119431290_119474250del). **B** Sanger sequencing of TRIM32 in the proband (c.1700A > G). **C** Multiplex sequence alignment in various species revealed that the variable base (567, labeled in red) was relatively conserved. **D** The predicted 3D models of the wild-type (right) and mutated (left) TRIM32 protein. An area different between the two was marked after contrastive analysis by PyMOL. **E** HEK 293 T cells transfected with a wild-type plasmid (pEGFP-TRIM32-WT) or mutant plasmids (pEGFP-TRIM32-D487N; pEGFP-TRIM32-H567R) under the same conditions showed that the mutant constructs exhibited diffuse cytoplasmic staining with loss of characteristic aggregates. Scale bar = 100 µm (× 40 magnification). **F** Western blot analysis of TRIM32 protein showed that the upper band corresponding to TRIM32/ubiquitin appeared in the wild-type and not in the mutant types (p.D487N and p.H567R). **G** Quantitation of TRIM32 gene transcription levels in the family

WGS analysis was used to predict the range of the deletion. Bioinformatics analysis based on WGS results predicted it to be approximately 46 kb in length. According to the relatively precise range estimation, primers on both sides of the breakpoints were designed, and unique gap-PCR products were obtained in II1 (Fig. 2A). Sanger sequencing revealed that the deletion (chr9.hg19:g.119431290_119474250del) was approximately 43 kb in size, with an additional insertion of nucleotide C. The deletion resulted in the removal of the whole TRIM32 gene in addition to a portion of ASTN2 downstream region. The transcription of ASTN2 gene was not affected because the promoter and exons were maintained intactly.

The novel missense mutation detected by NGS analysis was also confirmed by Sanger sequencing (Fig. 2B). This mutation caused the TRIM32 protein to change from histidine (H) to arginine (R) at position 567. The position was fully conserved across multiple species by species conservativeness analysis (Fig. 2C), and sequence analysis based on PolyPhen-2 predicted a score of 0.92, defined as possibly damaging. This mutation is located in the NHL domain of the TRIM32 protein. Structural modeling showed that the mutation changed the tertiary structure of the protein, causing the loss of portions of the alpha-helix.

### Functional analysis of the novel missense mutation

To evaluate the effect of the missense mutation, we transfected the plasmids into HEK 293 T cells and attempted to analyze the self-association ability and monoubiquitination of TRIM32 protein by confocal microscopy and western blot. The wild-type protein (TRIM32–WT) was observed with many aggregated spots in the cytoplasm, also called cytoplasmic bodies, and was usually located around the nucleus (Fig. 2E). Meanwhile, we found the transfected cells with cytoplasmic bodies contained monoubiquitinated TRIM32 by western blot (Fig. 2F). In contrast, the known pathogenic TRIM32-D487N mutant protein was observed to have diffused cytoplasmic staining with loss of characteristic aggregates and no ubiquitin. The TRIM32-H567R mutant caused by the novel missense mutation in this study also exhibited diffuse cytoplasmic staining similar to that of the TRIM32-D487N mutant, with the protein evenly dispersed in the cytoplasm and few characteristic aggregates(Fig. 2E). In addition, similar to the known TRIM32-D487N mutant, the TRIM32-H567R mutant also did not contain the protein ubiquitination that found in the wild-type protein (Fig. 2F).

Finally, we extracted RNA from the peripheral blood of the proband (II1) and her family members and then quantitatively analyzed the mRNA expression of TRIM32. Compared with normal controls, the TRIM32 transcript level was decreased by 80% in the proband, by 70% in her sister, and by only 50% in her brother, but her parents also decreased by approximately 60% (Fig. 2G).

### TRIM32 mutation spectrum and genotype–phenotype correlation in LGMD R8

**Fig. 3:**
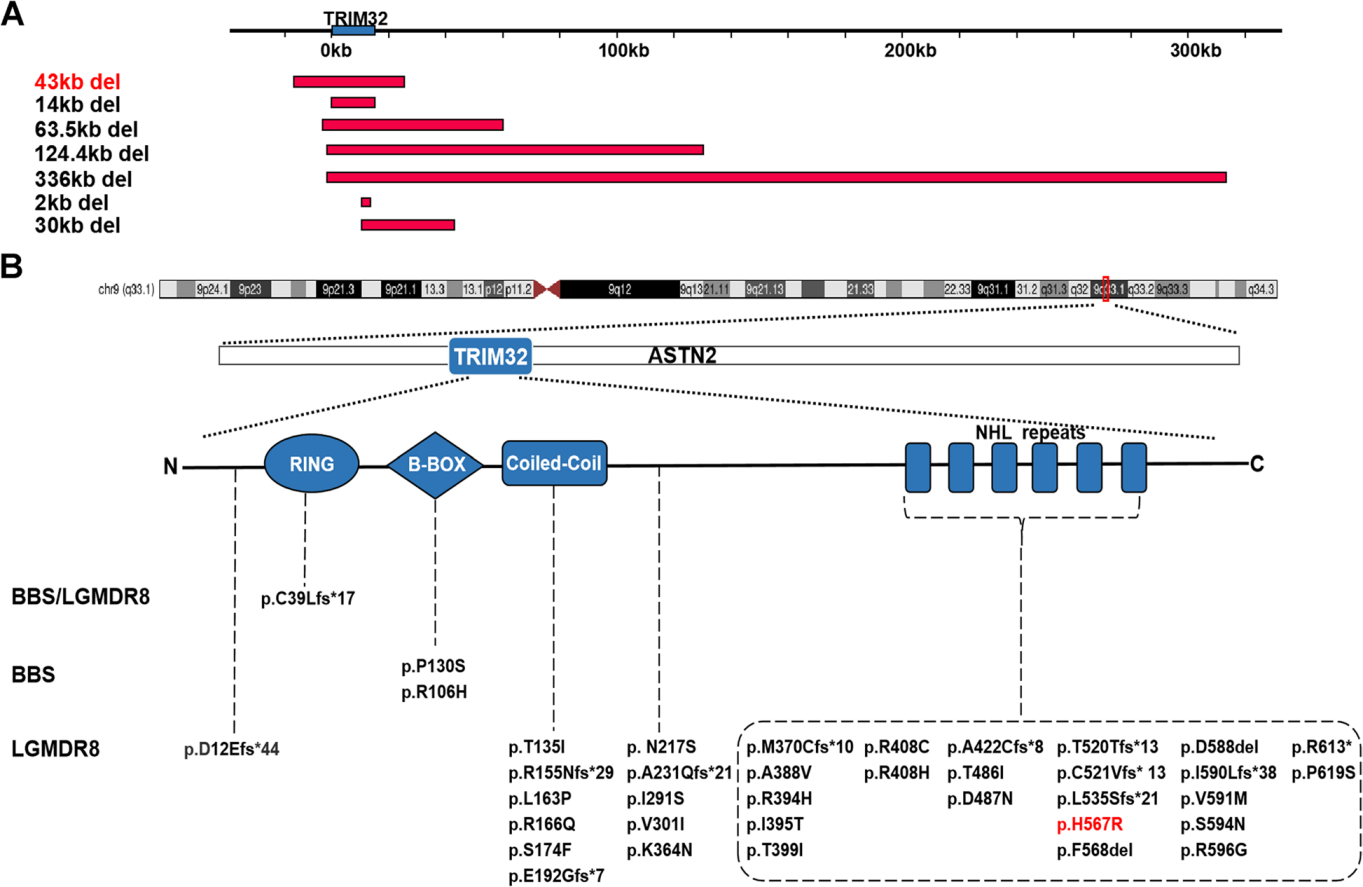
Literature review of TRIM32 mutations and phenotypes. **A** Schematic diagram of TRIM32 gene deletions (length and location).** B** Schematic diagram of the variants in the TRIM32 gene and the protein structure. TRIM32 is located on chromosome 9(q33.1), inlaid in the ASTN2 gene. All variants are arranged horizontally in five regions by location and vertically in 3 categories by disease. The mutation and deletion described in this article are marked in red

After searching the literature for TRIM32 mutations plus the newly identified mutations in this study, a total of 7 deletions (Fig. 3A) and 37 point mutations (Fig. 3B) were reported. All deletions resulted in the removal of the open reading frame of the TRIM32 gene. The shortest deletion was approximately 2 kb, and the longest deletion was approximately 336 kb. Among the 37 point mutations, there were 25 missense mutations, 11 shift mutations, and only 1 nonsense mutation. These mutations can be classified into five groups based on their location on the TRIM32 gene, of which one is located in the RING structural domain, two in the B-box structural domain, six in the coiled-coil structural domain, 22 in the NHL repeats, and the remaining 6 in intermediate regions outside the structural domain. We found that the vast majority of point mutations leading to LGMD R8 were clustered within the C-terminal NHL repeats (63% [22/35]), while those leading to BBS were located in the B-box structural domain.

From the published literature plus the two cases in this study, we collected 86 TRIM32 mutation-associated cases with detailed phenotype and genotype data (Supplementary Table S1). There were 22 male cases and 23 female cases, and the sex of the remaining 41 cases was not reported. Except for the hutterites, most patients were European, followed by Asian, and no patients of African were found. There were two cases of LGMD R8-merged BBS and one case of BBS, with a common feature of mutations that damage the B-box structure domain of TRIM32. The remaining 83 patients had LGMD R8, and five patients carrying monoallelic mutations showed only mild symptoms. The most common genotype was p.D487N homozygote, which was mainly distributed in the Canadian Hutterite population (51% [44/86]). The same genotype has significant phenotypic heterogeneity, with CK levels ranging from a low of 56 U/L to a high of 5556 U/L and cases ranging from asymptomatic to more severe clinical symptoms (such as being in a wheelchair).

**Fig. 4:**
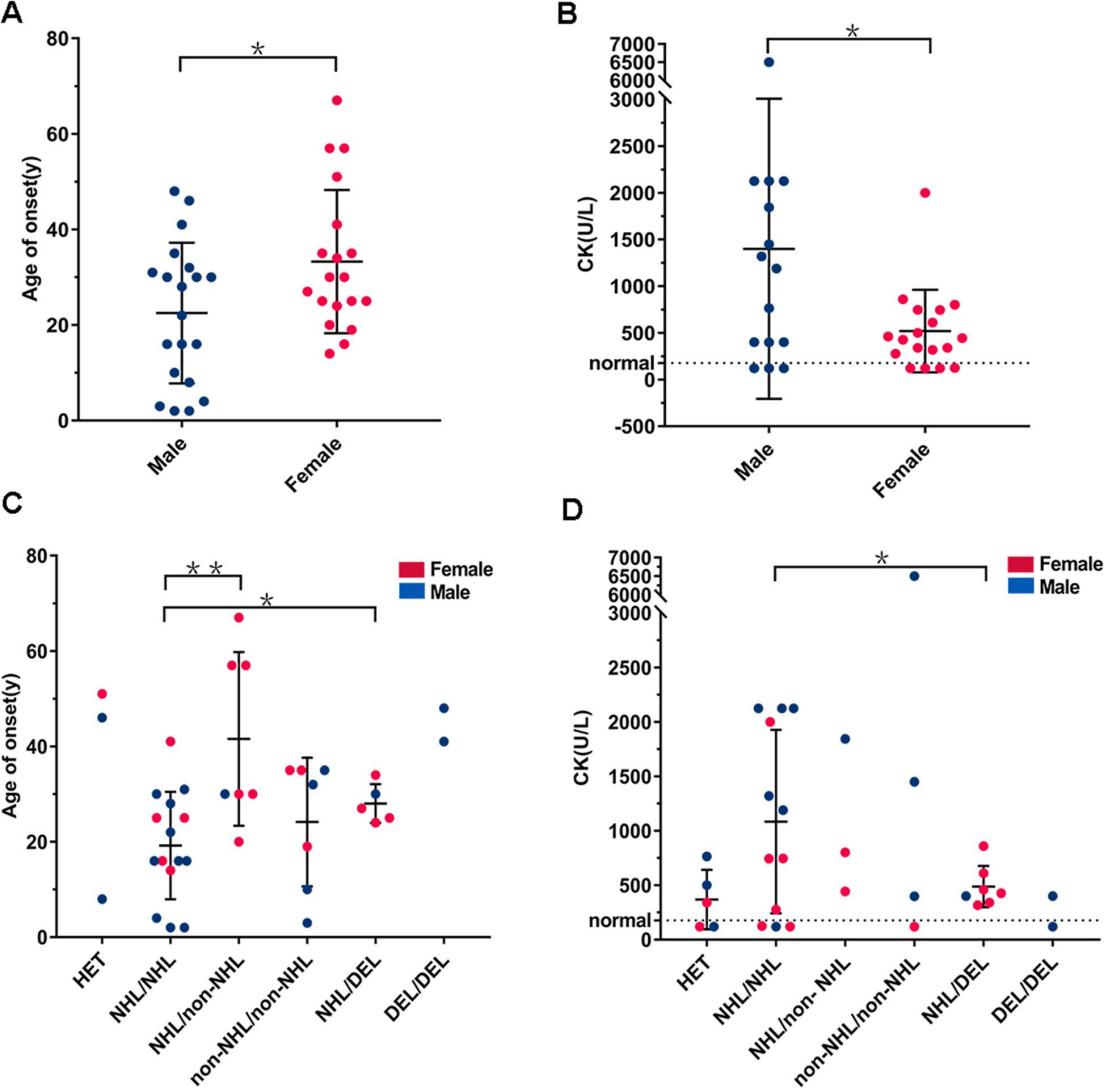
Cases carrying TRIM32 mutations reported by age of onset and CK level. **A**, **B** All TRIM32 alleles were classified into two types: male and female. **C**,** D** All TRIM32 alleles were classified into six types: HET (heterozygote), NHL/NHL (NHL repeats/NHL repeats), NHL/non-NHL (NHL repeats/non-NHL repeats), non-NHL/non-NHL (non-NHL repeats/non-NHL repeats), NHL/DEL (NHL repeats/deletion), and DEL/DEL (deletion/deletion). The dotted line shows the normal boundary line. Details and references for the cases in the figure can be seen in Supplementary Table S1

We divided the cases into two groups according to sex. It was observed that the age of onset in males was significantly lower than that in females (mean value: male 23 years old, female 33 years old, Fig. 4A, *p* < 0.05). Similarly, the CK levels of males were significantly higher than those of females (mean value: male 1400 U/L, female 519 U/L, Fig. 4B, *p* < 0.05). In addition, since most mutations causing LGMD R8 are clustered in the NHL repeat domain, we divided the cases into six groups according to the genotype of the TRIM32 gene, including heterozygote, NHL repeats/NHL repeats, non-NHL repeats/NHL repeats, non-NHL repeats/non-NHL repeats, NHL repeats/deletions, and deletions/deletions. As shown in Fig. 4C, we found that the cases in the NHL/NHL group had a significantly lower age of onset than those in the NHL/non-NHL (*p* < 0.01) and NHL/DEL groups (*p* < 0.05), but compared with the non-NHL/non-NHL group, the difference was not significant (*p* > 0.05). In terms of CK levels, compared to cases in the NHL/DEL group, cases in the NHL/NHL group had significantly higher CK levels (*p* < 0.05), but the cases with CK level data in the NHL/non-NHL group and non-NHL/non-NHL group were too few to compare (Fig. 4D).

## Discussion

In this study, a compound heterozygote composing a novel point mutation and a novel deletion of TRIM32 was identified in the LGMD R8 family members. We identified that the novel deletion of the patient was located at chr9.hg19:g.119431290_119474250. To date, six deletions have been reported (Fig. 3A), with the longest being 336 kb and shortest being 2 kb. In addition to including the entire TRIM32 gene, all 7 deletions also include part of the ASTN2 gene, which encodes astrotactin 2, a brain protein that may be involved in neuronal migration and is associated with neurodevelopmental disorders [20]. The longest deletion (336 kb del) includes a large portion of the ASTN2 gene, and the patient with this deletion shows mild progressive cognitive impairment in addition to LGMD R8 symptoms [21]. In addition, we confirmed that the patient had a missense mutation (TRIM32:c.1700A > G, p.H567R). The mutation occurred in the NHL repeat of the TRIM32 protein, a structural domain that primarily mediates self-association and interactions with its substrates. The location of the mutation was fully conserved in several species, and the mutation was predicted to be potentially deleterious. Compared with the wild-type protein, the mutant TRIM32 protein lost an alpha-helix at the end of the NHL repeats on the 3D image. Finally, we constructed this mutant vector and the wild-type vector for cell experiments and found a differential subcellular distribution. The wild-type protein was expressed as cytoplasmic spots with high fluorescence intensity, while the mutant (p.H567R) protein, similar to the previously reported p.D487N and p.R394H mutants, was expressed as diffuse cytoplasmic staining with loss of characteristic aggregation. In contrast, the p.P130S mutation, which was not located in the NHL repeat domain, was expressed as cytoplasmic spots similar to the wild-type. Patients with the P130S mutation also showed a BBS phenotype rather than LGMD R8 [22]. This indicated that the NHL repeats are the key region affecting the self-association of TRIM32 protein. According to the previous literature [10, 23], the function of ubiquitination of TRIM 32 protein is mainly rely on the self-association and the mutants lost this function. Western blot analysis (Fig. 2F) showed that the monoubiquitinated TRIM32 only present in wild-type, not in the known pathogenetic mutant (p.D487N) and our mutant (p.H567R). Therefore, the mutation in our study caused TRIM32 to lose its dimerization characteristics and be unable to participate in the degradation pathway of substrates, resulting in the occurrence of diseases [9]. In addition, we quantitatively analyzed TRIM32 mRNA in the blood of the proband (II1) and her family numbers and believed that the mRNA expression level does not reflect the phenotypic severity.

We collected 86 officially reported cases and performed the first analysis of LGMD R8 genotype–phenotype correlation. As summarized in Supplemental Table S1, there were 22 male cases and 23 female cases. The ratio of males to females is close to 1:1. However, the age of onset in males was significantly lower than that in females (Fig. 4A), and the CK value was significantly higher than that in females (Fig. 4B). These results indicated that although it is an autosomal disease, the condition of female patients is generally lighter than that of male patients. There have also been previous reports of earlier onset and earlier dependency on walking aids or wheelchairs in males than females with LGMDR2 [2, 24]. We speculate that the gender difference in the disease may be because males undertake more physical work in society, resulting in more severe muscle strain. However, it is noteworthy that the clinical data of female cases were always collected from patients who were not pregnant. The two Chinese female patients in our study also presented typical LGMD R8 symptoms. Nevertheless, they both were discovered through weakness and fatigue that proceeded during pregnancy. Each pregnancy in the proband resulted in significant deterioration of the condition, obstetric complications such as cesarean section, and no improvement in symptoms after delivery. In a retrospective study about pregnancy and delivery in women with myopathies [25], it was concluded that, compared with facioscapulohumeral muscular dystrophy (FSHD) patients, women with LGMD tend to have increased risk of aggravation of symptoms and obstetric complications. The increased risk of obstetric complications is closely related to muscle disease. Nine patients with LGMD were included in the research whose gene diagnosis was not available due to the limitation of molecular diagnosis at that time [25]. Nowadays a sophisticated classification system of LGMD has been established and pathogenetic mutation could be detected in most LGMD patients. We think it feasible and worthwhile to perform detailed neurological analysis throughout the procedure of pregnancy of women with different genetic subgroup of LGMD. The results will be instructive for providing precise support for this special population, from pre-pregnancy assessment to pregnancy monitoring plan, prevention and treatment of obstetric complications, delivery mode selection, and postpartum rehabilitation guidance.

Finally, we summarized the known deletions and point mutations in the TRIM32 gene, with point mutations accounting for the majority. There are no hotspot mutations, and the population frequency is very low, less than 1 in 10,000. We classified the point mutations by the structural domain of the protein and found that the point mutation leading to BBS was located in the B-box, while most of the point mutations leading to LGMD R8 were located in NHL repeats. Similar to the OCRL gene previously reported, the mutation will not only cause Dent disease type 2 (DD2) but also cause Lowe syndrome (LS) involving the eyes, brain and kidney. The location of DD2 mutations in OCRL is quite different from that of LS mutations, since the majority of DD2 pathogenic variants mapped within the 5′ region, while those responsible for the LS phenotype mapped in the 3′ region of the gene [26]. Therefore, we suggest that the point mutation site of TRIM32 determines the LGMD R8 phenotype and the NHL repeats are the key point of LGMD R8 pathogenesis. We classified the cases by mutation site and genotype. Most of the point mutations have a lower age of onset and higher CK levels than the deletions (Fig. 4C, D), possibly because the faulty protein affects the action of other proteins and is more harmful than the absolute reduction of normal proteins [27]. More importantly, we found that the patients carrying two point mutations in the NHL repeats of TRIM32 protein were the most common and had a lower age of onset than other patients. Simultaneously, this structural domain mainly mediates the self-association of TRIM32, so we believe that the degree of dimerization of this protein is related to the severity of the disease. If the relationship between them is further investigated, the correlation between genotype and phenotype can be better described, providing better opinions for future diagnosis and treatment.

## Conclusions

In conclusion, we identified the first Chinese patient with LGMD R8 caused by a missense mutation in compound heterozygosis with a deletion of TRIM32 and showed that the novel mutation is pathogenic by functional analysis. Furthermore, the mutational spectrum of TRIM32 was reviewed, and a first preliminary analysis of LGMD R8 genotype–phenotype correlations was performed. Our study expands the range of TRIM32 gene mutations and has important implications for future molecular diagnosis, therapy, and genetic counseling.

## Supplementary Information

**Additional file 1: Supplemental Table S1.** The clinical and genetic information of TRIM32 mutation cases.

## Data Availability

All data generated or analyzed during this study are included in this published article [and its supplementary information files].

## Abbreviations

NMD: Neuromuscular diseases
LGMD R8: Limb-girdle muscular dystrophy R8
CK: Creatine kinase
STM: Sarcotubular myopathy
BBS: Bardet Biedl syndrome
NGS: Next-generation sequencing
WGS: Whole genome sequencing
HEK: Human embryonic kidney
EMG: Electromyography
MRI: Magnetic resonance imaging
MUAPs: Motor unit action potentials
NCSs: Nerve conduction studies
DD2: Dent disease type 2
LS: Lowe syndrome
