# Supplementary material for: TRIM32 biallelic defects cause limb-girdle muscular dystrophy R8: identification of two novel mutations and investigation of genotype–phenotype correlation
Source: Skelet Muscle. 2023 May 22;13:10. doi: 10.1186/s13395-023-00319-x (PMC10201696; doi:10.1186/s13395-023-00319-x)
Supplement: Supplementary file 1 — Additional file 1: Supplemental Table S1. The clinical and genetic information of TRIM32 mutation cases. [file 13395_2023_319_MOESM1_ESM.docx]

**Supplemental Table 1.**The clinical and genetic information of TRIM32 mutation cases

| **ID** | **Sex** | **Region** | **Clinical information** | | | **Genetic information** | | **Reference** |
| --- | --- | --- | --- | --- | --- | --- | --- | --- |
|  |  |  | **Age of onset(y)** | **CK(U/L)** | **Phenotype** | **Genotype** | **Region Class** |  |
| 1 | M | Italy | n.r. | 500 | Asymptomatic hyper-CKemia | p.R394H/N | Het | PMID: 17994549 |
| 2 | M | Europe | 8 | 765 | LGMDR8 | p.D588del/N | Het | PMID: 17994549 |
| 3 | F | Sweden | 51 | Normal(120) | Mild LGMDR8 | p.C521Vfs* 13/N | Het | PMID: 19492423 |
| 4 | F | Sweden | birth | 340 | Mild LGMDR8 | 30 kb deletion/N | Het | PMID: 19492423 |
| 5 | M | Sweden | 46 | Normal(120) | Mild LGMDR8 | 30 kb deletion/N | Het | PMID: 19492423 |
| 6 | F | Spain | 35 | n.r. | LGMDR8，BBS | p.C39Lfs*17/p.C39Lfs*17 | non-NHL/non-NHL | [PMID: 30823891](https://www.ncbi.nlm.nih.gov/pubmed/30823891) |
| 7 | M | Spain | 35 | n.r. | LGMDR8，BBS | p.C39Lfs*17/p.C39Lfs*17 | non-NHL/non-NHL | [PMID: 30823891](https://www.ncbi.nlm.nih.gov/pubmed/30823891) |
| 8 | F | Spain | 35 | n.r. | LGMDR8 | p.C39Lfs*17/p.C39Lfs*17 | non-NHL/non-NHL | [PMID: 30823891](https://www.ncbi.nlm.nih.gov/pubmed/30823891) |
| 9 | M | Arab | n.r. | n.r. | BBS | p. P130S/p. P130S | non-NHL/non-NHL | [PMID: 16606853](https://www.ncbi.nlm.nih.gov/pubmed/16606853) |
| 10 | M | Turkey | 10 | 1450 | LGMDR8 | p.R155Nfs*29/p.R155Nfs*29 | non-NHL/non-NHL | [PMID: 29921608](https://www.ncbi.nlm.nih.gov/pubmed/29921608) |
| 11 | M | Pakistan | 3 | 6500 | LGMDR8 | p.L163P/p.L163P | non-NHL/non-NHL | [PMID: 29921608](https://www.ncbi.nlm.nih.gov/pubmed/29921608) |
| 12 | M | Persia | 32 | 398 | LGMDR8 | p.L163P/p.L163P | non-NHL/non-NHL | [PMID: 29921608](https://www.ncbi.nlm.nih.gov/pubmed/29921608) |
| 13 | F | Persia | 19 | normal(120) | LGMDR8 | p.I291S/p.I291S | non-NHL/non-NHL | [PMID: 29921608](https://www.ncbi.nlm.nih.gov/pubmed/29921608) |
| 14 | F | Belgium | 30 | 443 | LGMDR8 | p.E192Gfs*7/p.A388V | non-NHL/NHL | [PMID: 29921608](https://www.ncbi.nlm.nih.gov/pubmed/29921608) |
| 15 | F | Belgium | 30 | 802 | LGMDR8 | p.E192Gfs*7/p.A388V | non-NHL/NHL | [PMID: 29921608](https://www.ncbi.nlm.nih.gov/pubmed/29921608) |
| 16 | F | Australia | 20 | n.r. | LGMDR8 | p. N217S/p.F568del | non-NHL/NHL | [PMID: 30823891](https://www.ncbi.nlm.nih.gov/pubmed/30823891) |
| 17 | F | Australia | 57 | n.r. | LGMDR8 | p. N217S/p.F568del | non-NHL/NHL | [PMID: 30823891](https://www.ncbi.nlm.nih.gov/pubmed/30823891) |
| 18 | F | Australia | 57 | n.r. | LGMDR8 | p. N217S/p.F568del | non-NHL/NHL | [PMID: 30823891](https://www.ncbi.nlm.nih.gov/pubmed/30823891) |
| 19 | F | Australia | 67 | n.r. | LGMDR8 | p. N217S/p.F568del | non-NHL/NHL | [PMID: 30823891](https://www.ncbi.nlm.nih.gov/pubmed/30823891) |
| 20 | M | Britain | 30 | 1844 | LGMDR8 | p.A231Qfs*21/p.M370Cfs*10 | non-NHL/NHL | [PMID: 29921608](https://www.ncbi.nlm.nih.gov/pubmed/29921608) |
| 21 | M | Italy | 30 | n.r. | LGMDR8 | p.R394H/p.R394H | NHL/NHL | PMID: 17994549 |
| 22 | F | Netherlands | 41 | 125 | LGMDR8 | p.I395T/p.I395T | NHL/NHL | PMID: 30919934 |
| 23 | F | Serbian | 25 | 276 | LGMDR8 | p.D487N/p.D487N | NHL/NHL | [PMID: 29921608](https://www.ncbi.nlm.nih.gov/pubmed/29921608) |
| 24 | M | Hutterite | 2 | normal(120) | LGMDR8 | p.D487N/p.D487N | NHL/NHL | PMID:15786463 |
| 25 | M | Hutterite | 2 | 5-20n(2125) | LGMDR8 | p.D487N/p.D487N | NHL/NHL | PMID:15786463 |
| 26 | M | Bosnian Serb | 28 | 1189 | LGMDR8 | p.D487N/p.D487N | NHL/NHL | [PMID: 29921608](https://www.ncbi.nlm.nih.gov/pubmed/29921608) |
| 27 | M | Hutterite | 22 | 1320 | LGMDR8 | p.D487N/p.D487N | NHL/NHL | PMID: 23142638 |
| 28 | M | Germany | 31 | 5-20n(2125) | LGMDR8 | p.D487N/p.D487N | NHL/NHL | PMID:15786463 |
| 29 | M | Germany | 4 | 5-20n(2125) | LGMDR8 | p.D487N/p.D487N | NHL/NHL | PMID:15786463 |
| 30-70 | n.r. | Hutterite | 9-42(24) | 81-5556 | LGMDR8 | p.D487N/p.D487N | NHL/NHL | PMID: 15580560 |
| 71 | F | Croatian | n.r. | normal(120) | LGMDR8 | p.T520Tfs'13/p.T520Tfs'13 | NHL/NHL | PMID: 17994549 |
| 72 | F | Turkey | 25 | 744 | LGMDR8 | p.I590Lfs*38/p.I590Lfs*38 | NHL/NHL | PMID: 19303295 |
| 73 | M | Spain | teenager(16) | n.r. | LGMDR8 | p.V591M/p.V591M | NHL/NHL | [PMID: 30823891](https://www.ncbi.nlm.nih.gov/pubmed/30823891) |
| 74 | M | Spain | teenager(16) | n.r. | LGMDR8 | p.V591M/p.V591M | NHL/NHL | [PMID: 30823891](https://www.ncbi.nlm.nih.gov/pubmed/30823891) |
| 75 | M | Spain | teenager(16) | n.r. | LGMDR8 | p.V591M/p.V591M | NHL/NHL | [PMID: 30823891](https://www.ncbi.nlm.nih.gov/pubmed/30823891) |
| 76 | F | Italy | 14 | ≤2000 | LGMDR8 | p.R596G/p.R596G | NHL/NHL | [PMID: 29921608](https://www.ncbi.nlm.nih.gov/pubmed/29921608) |
| 77 | F | Azerbaijani | teenager(16) | 746 | LGMDR8 | p.P619S/p.P619S | NHL/NHL | [PMID: 34439639](https://www.ncbi.nlm.nih.gov/pubmed/34439639) |
| 78 | F | Poland | 34 | 317 | LGMDR8 | p.R613*/63.5 kb deletion | NHL\DEL | [PMID: 29921608](https://www.ncbi.nlm.nih.gov/pubmed/29921608) |
| 79 | F | Sweden | <10 | 460 | LGMDR8 | p.C521Vfs* 13/30 kb deletion | NHL\DEL | PMID: 19492423 |
| 80 | F | Sweden | <10 | 860 | LGMDR8 | p.C521Vfs* 13/30 kb deletion | NHL\DEL | PMID: 19492423 |
| 81 | M | n.r. | 30 | 400 | LGMDR8 | p.L535Sfs*21/124.4kb deletion | NHL\DEL | [PMID: 25351777](https://www.ncbi.nlm.nih.gov/pubmed/25351777) |
| 82 | F | China | 24 | 610 | LGMDR8 | p.H567R/43kb deletion | NHL\DEL | our cases |
| 83 | F | China | 27 | 427 | LGMDR8 | p.H567R/43kb deletion | NHL\DEL | our cases |
| 84 | F | n.r. | 25 | 340 | LGMDR8 | p.R613*/Entire deletion | NHL\DEL | PMID: 23541687 |
| 85 | M | China | 48 | 400 | LGMDR8 | 2kb deletion/2kb deletion | DEL\DEL | [PMID: 33485293](https://www.ncbi.nlm.nih.gov/pubmed/33485293) |
| 86 | M | n.r. | 41 | normal(120) | LGMDR8 | 336kb deletion/336kb deletion | DEL\DEL | [PMID: 25351777](https://www.ncbi.nlm.nih.gov/pubmed/25351777) |

F=female; M=male; CK=creatine kinase(reference range 2-178 IU/L); n.d.= not done; n.r.=not reported; N=normal allele ;HET=Heterozygote; NHL=NHL repeats; non-NHL=non-NHL repeats; DEL=deletion
